# The impact of intravenous methylprednisolone pulses on renal survival in anti-neutrophil cytoplasmic antibody associated vasculitis with severe renal injury patients: a retrospective study

**DOI:** 10.1186/s12882-017-0782-4

**Published:** 2017-12-29

**Authors:** Yanhong Ma, Fei Han, Liangliang Chen, Hongya Wang, Haidongqing Han, Binfeng Yu, Ying Xu, Jianghua Chen

**Affiliations:** 1 Kidney Disease Center, First Affiliated Hospital, College of Medicine, Zhejiang University, Hangzhou, People’s Republic of China; 2 Key Laboratory of Kidney Disease Prevention and Control Technology, 79 Qingchun Road, Hangzhou, Zhejiang Province 310003 People’s Republic of China; 3 Third Grade Laboratory under the National State Administration of Traditional Chinese Medicine, Hangzhou, People’s Republic of China

**Keywords:** Anti-neutrophil cytoplasmic autoantibody, Vasculitis, Methylprednisolone, Kidney, Outcome

## Abstract

**Background:**

High-dose methylprednisolone pulses were one of the main treatments for anti-neutrophil cytoplasmic autoantibody (ANCA) associated vasculitides (AAV) but had obvious side effects. We aimed to know the impact on renal survival and identify the prognostic factors of this treatment in Chinese AAV patients with severe renal involvement.

**Methods:**

One hundred and eleven AAV patients with an estimated glomerular filtration rate (eGFR) of 10ml/min/1.73 m^2^ or less at admission were included. The MP group (*n* = 57) received intravenous methylprednisolone 500 mg/d for 3 days, while the control group (*n *= 54) had not. The outcomes and adverse events between two groups were compared. Besides, predictors for dialysis independence and good response of intravenous methylprednisolone were analyzed using Cox regression analysis and ROC curves respectively.

**Results:**

Their median duration of follow-up was 31 (range 3 to 134) months. Eleven patients in MP group and 20 patients in control group were died (*P* = 0.056). Twenty-one patients (36.8%) in MP group and 29 patients (53.7%) in control group were on maintaining dialysis (*P *= 0.088). Twenty-one patients in MP group remained dialysis independent, more than those in control group (4 patients, *P *<0.01). Urine protein creatinine ratio (hazard ratio 1.730, 95% confidence interval 1.029 to 2.909, *P* = 0.039) and the treatment of intravenous methylprednisolone pulses (hazard ratio 0.362, 95% confidence interval 0.190 to 0.690, *P* = 0.002) were the independent risk factors for dialysis independence. Those patients with serum creatinine≥855μmol/L and urine protein ≥3.7g/24h at admission may have worse responses to intravenous methylprednisolone pulses (sensibility 56.7%, specificity 85.0%, PPV 100.0% and NPV57.1%).

**Conclusions:**

Intravenous methylprednisolone pulses could improve the long-term outcome in term of dialysis independence and tend to decrease mortality for Chinese AAV patients with severe renal involvement.

**Electronic supplementary material:**

The online version of this article (10.1186/s12882-017-0782-4) contains supplementary material, which is available to authorized users.

## Background

Anti-neutrophil cytoplasmic auto-antibody (ANCA) associated vasculitides (AAV) is characterized by inflammation in small vessels and the presence of auto-antibodies directing against lysosomal components of neutrophils and monocytes [1], including granulomatosis with polyangiitis (GPA), microscopic polyangiitis (MPA) and eosinophilic granulomatosis with polyangiitis (EGPA) [2–5]. AAV commonly affects kidney and lung [6]. In Chinese population, nearly 90% of AAV cases had renal involvement [7], presenting as pauci-immune focal and segmental necrotizing and crescentic glomerulonephritis (NCGN), a syndrome signified by a precipitous loss of renal function, with features of glomerulonephritis including dysmorphic erythrocyturia and glomerular proteinuria [8]. Berden et al suggested four general categories of renal pathological lesions (focal, crescentic, mixed, and sclerotic lesions) of ANCA associated glomerulonephritis; they validated these categories in 100 biopies from 61 MPA patients and 39 GPA patients in European countries and found crescentic lesions (more than 50% glomeruli with celular crescents) accounted for 55% in these patients [9]. The published data in Japanese and Chinese populations reported that AAV patients in these populations had higher percentage of MPA (79–93% in Japanese or Chinese population, whereas 44–69% in European population) and were older than western populations [7, 10–12]. They also had higher percentages of sclerotic and mixed lesions in renal pathology and may have worse renal outcomes [13–15].

It was reported that up to 30% of AAV patients with renal involvement eventually developed end-stage renal disease (ESRD) [16, 17]. Kidney Disease: Improving Global Outcomes (KDIGO) guidelines recommended cyclophosphamide and corticosteroids as initial treatments. The high-dose methylprednisolone pulses were one of the main treatments for induction therapy. However the rates of severe adverse events following pulse methylprednisolone were high, in particular severe infections, which were the main causes for death [18]. Jayne et al reported that about 43% of AAV patients progressed to ESRD and 28% patients died of infections in 12 months using high-dose methylprednisolone as adjunctive therapy for severe renal vasculitis [18]. Poor outcomes in AAV patients may be caused by disease progression due to lack of intensive immunosuppressive treatment or severe side effects of immunosuppressive treatments [19]. It is important to know the kind of patients who need intensive immunosuppressive treatment and who will have good responses to the intensive treatment. In this retrospective study, we analyzed the effects of high-dose methylprednisolone pulses in Chinese AAV patients with severe renal injury and aimed to know the factors that may affect the effects of methylprednisolone pulses.

## Methods

We retrospectively investigated the AAV patients who were diagnosed and followed up in the Kidney Disease Center of First Affiliated Hospital, College of Medicine, Zhejiang University from January 2004 to June 2016. The end of follow-up date was September 30, 2016. AAV was diagnosed and classified according to 2012 Chapel Hill Consensus Conference on nomenclature of vasculitides [2] and a previous European Union study [20]. Patients who had an estimated glomerular filtration rate (eGFR) of 10ml/min/1.73 m^2^ or less at admission were included [21]. Cases secondary to the diseases including IgA nephropathy, systemic lupus erythematosus, anti-glomerular basement membrane (GBM) antibody disease, post-infectious glomerulonephritis, Henoch-Schonlein purpura, cryoglobulinemic vasculitis, membranous and membranoproliferative glomerulonephritis were excluded by the clinical and pathologic presentations. The study protocols conformed to the provisions of the Declaration of Helsinki and were approved by the Ethic Committee of the First Affiliated Hospital of Medical School of Zhejiang University (reference number: 201721).

All patients followed a standard treatment including prednisone (1mg/kg/d) or prednisone (0.6-0.8mg/kg/d) combined with intravenous cyclophosphamide (0.75-1.0g/m^2^ in monthly pulses) or prednisone (0.6 to 0.8mg/kg/d) combined with mycophenolate mofetil (1.0-1.5g/d). The MP group received intravenous methylprednisolone 500 mg/d for 3 days. The control group had no intravenous methylprednisolone pulses. All the patients had no plasma exchange.

The clinical data included demographic characteristics, clinical presentations, histopathologic changes, laboratory parameters, treatments and outcomes. The estimated glomerular filtration rate (eGFR) was calculated by modification of diet in renal disease equations for Chinese patients [22]. The serum ANCA level was measured by indirect IF and titrated by antigen-specific ELISA using myeloperoxidase (MPO) or proteinase 3 (PR3) as the antigens. Urine protein creatinine ratio (UPCR) was calculated from testing of urine for protein and creatinine using spot urinary samples. Disease activity was measured by the Birmingham Vasculitis Activity Score (BVAS) [23]. Kidney length was defined as the mean value of the lengths of bilateral kidneys tested by ultrasound since no patient had solitary kidney or unilateral renal atrophy. The pathologic specimens for light microscopy were fixed in 4.5% formaldehyde solution. The pathologic changes such as global glomerular sclerosis, crescent formation, glomerular fibrinoid necrosis, interstitial fibrosis and interstitial inflammation were analyzed. The severity of mesangial proliferation was semi-quantitatively scored as 1/2/3 and the severity of interstitial infiltrates was semi-quantitatively scored as 0/1/2/3. All the renal biopsy specimens were re-evaluated by two observers. The outcome of maintaining dialysis was defined by dialysis requirement for at least 3 months without subsequent renal recovery [18].

The software used for statistical analyses was the SPSS version 23.0. Most numerical results were expressed as mean±SD. Data for global glomerular sclerosis and crescent formation were summarized as the median and interquartile range (IQR). Differences of numerical data with normal distribution were tested by Student’s t test. Other numerical data, semi-quantitative scores were compared by Mann-Whitney U-test. Categorical data were interpreted in the form of constituent ratio and percentage, and compared by χ^2^ test. The Mann-Whitney tests were used for nonparametric comparisons. A P value<0.05 was considered significant unless otherwise stated. For assessment of risk factors for outcomes, univariate analysis and multivariate Cox regression analysis were conducted. Each parameter that correlated with P≤0.1 was stratified and entered in the Cox model. For assessment of possible predictors for response of intravenous methylprednisolone pulses, ROC curves were analyzed and the cut-off value was based on the maximum of Youden index (sensitivity + specificity - 1).

## Results

We screened 492 AAV patients and excluded 363 patients with eGFR greater than 10ml/min/1.73 m^2^, 7 patients with AAV secondary to other known diseases (IgA nephropathy, anti-glomerular basement membrane (GBM) antibody disease, post-infectious glomerulonephritis, Henoch-Schonlein purpura, cryoglobulinemic vasculitis (one case each), systemic lupus erythematosus (two cases)), 5 patients treated without prednisone and 6 patients treated with plasma exchange. Totally 111 AAV patients were included in this study. There were 57 patients in MP group and 54 patients in control group. The yearly numbers of patients in MP group and control group enrolled from 2004 to 2016 were shown in figure S1 in the supplemental file. Their median duration of follow-up in our center was 31 (range, 3 to 134) months. Five patients were lost to follow up, including 4 patients in MP group (one patient was lost on 8th month, three patients were lost on 3rd month) and one patient in control group (on 3rd month).

### Baseline characteristics

The baseline clinical characteristics were shown in Table 1. There were no significant differences in demographic and laboratory results at baseline between MP group and control group. Only the ultrasonic kidney length was significant less in control group than that in MP group. The serum creatinine and eGFR levels were 654.0 ± 202.7μmol/L and 7.0 ± 1.9ml/min/1.73m^2^ in MP group, 715.9 ± 171.0μmol/L and 6.5 ± 1.8ml/min/1.73m^2^ in control group (*P* = 0.086, *P* = 0.147 respectively). Most of the patients were MPO-ANCA positive (89.5% in MP group and 94.4% in control group). The BVAS was equal in both groups (15.2 ± 3.0 in MP group and 15.4 ± 2.9 in control group). Only 34 patients had kidney biopsy results (28 biopsies in MP group and 6 biopsies in control group). The renal pathology was compared between MP group and control group in Table S1 in the supplemental file. There were no significant differences in proportions of crescents, glomerular global sclerosis or fibrinoid necrosis between MP group and control group. However as the number of biopsies was few and the distribution between MP group and control group was unbalanced, it was hard to assess the overall pathological significance.

**Table 1:** The baseline clinical characteristics in MP group and control group

	MP group (*n* = 57)	Control group (*n* = 54)	*P* value
Age (year)	56.9 ± 12.7	60.5 ± 12.3	0.206
Female (n, %)	41, 73.7	30, 55.6	0.079
White blood cells (10^9^/L)	9.0 ± 4.8	7.8 ± 3.6	0.144
Hemoglobin (g/L)	73.5 ± 13.1	74.1 ± 13.8	0.824
Platelet (10^9^/L)	211.2 ± 84.7	191.4 ± 93.4	0.244
Serum albumin (g/L)	31.1 ± 5.0	32.1 ± 5.3	0.282
Serum globulin (g/L)	30.3 ± 5.2	29.2 ± 6.2	0.282
Alanine transaminase (u/L)	14.7 ± 12.2	12.2 ± 8.1	0.205
Serum creatinine (μmol/L)	654.0 ± 202.7	715.9 ± 171.0	0.086
≥500 μmol/L (case, %)	50, 92.6	46, 80.7	0.095
eGFR (ml/min/1.73 m^2^)	7.0 ± 1.9	6.5 ± 1.8	0.147
UPCR (g/24 h)	3.1 ± 1.7	3.4 ± 2.6	0.423
≥3.5g/24h (case, %)	15, 27.8	18, 35.3	0.528
ESR (mm/h)	89.9 ± 38.0	75.3 ± 37.3	0.057
CRP (mg/L)	43.2 ± 49.0	37.0 ± 44.4	0.498
MPO-ANCA positive (case, %)	51, 89.5	51, 94.4	0.491
PR3-ANCA positive (case, %)	7, 12.3	3, 5.6	0.322
Pulmonary involvement (case, %)	42, 73.7	39, 72.2	1.000
BVAS	15.2 ± 3.0	15.4 ± 2.9	0.681
Kidney length (cm)	10.7 ± 1.1	9.8 ± 1.4	0.001
P/P+IVC/P+MMF/P+others (case)	7/28/21/1	14/21/16/3	0.177

*eGFR* Estimated glomerular filtration rate, *UPCR* Urine protein creatinine ratio, *ESR* Erythrocyte sedimentation rate, *CRP* C reactive protein, *MPO* myeloperoxidase, *PR3* Proteinase 3, *BVAS* Birmingham Vasculitis Activity Score, *IVC* Intravenous cyclophosphamide, *MMF* Mycophenolate mofetil, *P* Prednisone

### Outcomes and adverse events

**Fig. 1:**
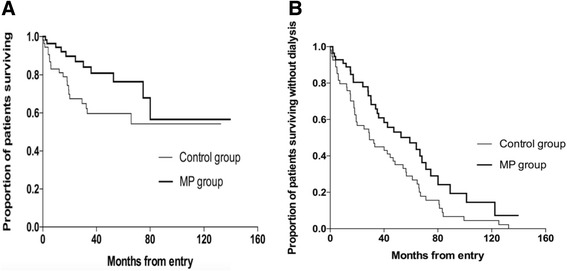
**a** Proportion of patients in each group who survived during the study intravenous methylprednisolone group [MP] *versus* control group Log Rank = 3.428, *P* = 0.064). **b** Proportion of patients in each group who survived without dialysis (MP *versus* control group Log Rank = 30.299, *P* <0.01).

The outcome events of MP group and control group were shown in Table 2. By the end of observation, 11 patients in MP group and 20 patients in control group were died (*P* = 0.056); the major causes of death were infection (22 cases), cardiovascular diseases (4 cases), hemorrhage (3 cases) and cancer (2 cases). Twenty-one patients (36.8%) in MP group and 29 patients (53.7%) in control group were on maintaining dialysis (*P* = 0.088). Twenty-one patients in MP group remained dialysis independent, more than those in control group (4 patients, P <0.01). The survival curves of MP group and control group were shown in Fig. 1. The patient survival was 80.7% in MP group and 63.0% in control group (Log Rank = 3.428, *P* = 0.064). The dialysis free survival was 36.8% in MP group and 7.4% in control group (Log Rank=30.299, *P* <0.01).

**Table 2:** The patient outcomes in MP group and control group

	MP group (*n* = 57)	Control group (*n* = 54)	*P* value
Follow up duration (month, medium (range))	32 (3-140)	29 (3-133)	0.922
Serum creatinine (median (IQR), μmol/L)	383.0 (233.5-548.0)	700.0 (436.8-830.5)	<0.01
eGFR (median (IQR), ml/min/1.73 m^2^)	13.0 (8.0-23.8)	7.1(5.3-9.8)	<0.01
Death	11, 19.3%	20, 37.0%	0.056
Infections	9	13	
Cardiovascular diseases	1	3	
Hemorrhage	0	3	
Cancer	1	1	
Maintaining dialysis	21, 36.8%	29, 53.7%	0.088
Dialysis independent	21, 36.8%	4, 7.4%	<0.01
CKD-II	1	0	
CKD-III	4	2	
CKD-IV	7	0	
CKD-V	9	2	
Lost	4, 7.0%	1, 1.3%	0.364

A total of 123 adverse events were reported in MP group and control group, shown in Table 3. The rates of adverse events were similar between MP group and control group. Infections, thrombocytopenia and leukopenia were most common in both groups.

**Table 3:** Adverse events in MP group and control group

Adverse events	MP group (*n* = 57)	Control group (*n* = 54)	*P* value
Infection	28	26	1.000
bacteria	19	18	1.000
fungus	4	3	1.000
virus	5	5	1.000
Thrombocytopenia	13	9	0.480
Leukopenia	8	5	0.559
Diabetes	4	5	0.738
Gastrointestinal	4	4	1.000
Cardiovascular	2	5	0.263
Hemorrhage	3	3	1.000
Bone fracture	0	1	0.486
Vascular access complication	0	1	0.486
Epilepsy	1	0	1.000
Cataract	0	1	0.486
Totals	63	60	

### Risk factors for outcomes

We reclassified all the 106 patients (exclude 5 lost patients) into two groups according to the outcomes. Twenty-five patients with the outcome of dialysis independence were included in one group while 81 patients with the outcome of death or maintaining dialysis were included in the other group. The characteristics in both groups were compared in Table 4. The patients in the group of death or maintaining dialysis had higher serum creatinine level and more urine protein. The percentage of intravenous methylprednisolone pulses in the group of death or maintaining dialysis was significant less than that in dialysis independent group (39.5% vs 84%, *P* <0.001). We included the rate of kidney biopsy, the kidney length and the factors with *P* value less than 0.1 (gender, serum creatinine, UPCR, eGFR and the treatment of intravenous methylprednisolone pulses) into Cox regression analysis, setting death or maintaining dialysis as the defined event. We found that UPCR (hazard ratio 1.730, 95% confidence interval 1.029 to 2.909; *P* = 0.039) and intravenous methylprednisolone pulses (hazard ratio 0.362, 95% confidence interval 0.190 to 0.690; *P*=0.002) were identified as independent risk factors.

**Table 4:** The characteristics in dialysis independent group and death or maintaining dialysis group according to the patient outcomes

Characteristics	Dialysis independent group (*n* = 25)	Death/ maintaining dialysis group (*n* = 81)	*P* value
Age (year)	59.5 ± 12.7	59.3 ± 15.1	0.950
Female (n, %)	20, 80.0	46, 56.8	0.058
White blood cells (10^9^/L)	9.4 ± 4.6	8.0 ± 4.2	0.152
Hemoglobin (g/L)	74.0 ± 15.7	73.8 ± 12.7	0.969
Platelet (10^9^/L)	225.2 ± 101.4	193.2 ± 86.1	0.122
Serum albumin (g/L)	31.5 ± 5.7	31.7 ± 5.0	0.907
Serum globulin (g/L)	30.5 ± 7.0	29.7 ± 5.3	0.554
Alanine transaminase (u/L)	14.8 ± 13.4	12.4 ± 7.3	0.392
Serum creatinine (μmol/L)	601.1 ± 150.3	718.4 ± 193.8	0.007
≥500 μmol/L (case, %)	18, 72.0	75, 92.6	0.012
eGFR (ml/min/1.73m^2^)	7.2 ± 1.9	6.5 ± 1.8	0.097
UPCR (g/24 h)	2.5 ± 0.7	3.4 ± 2.4	0.004
≥3.5g/24h (case, %)	3, 12.5	27, 35.5	0.041
ESR (mm/h)	83.0 ± 42.3	81.5 ± 37.1	0.875
CRP (mg/L)	48.0 ± 53.8	38.8 ± 45.2	0.401
MPO-ANCA positive (case, %)	22, 88.0	73, 92.4	0.446
PR3-ANCA positive (case, %)	4, 16.0	6, 7.7	0.251
Pulmonary involvement (case, %)	18, 72.0	61, 75.3	0.795
BVAS	14.6 ± 3.2	15.6 ± 2.8	0.174
Kidney length (cm)	10.6 ± 1.1	10.1 ± 1.4	0.113
Infection (case, %)	10, 40.0	31, 38.3	1.000
Intravenous methylprednisolone pulses (case, %)	21, 84.0	32, 39.5	<0.001
Kidney biopsy (case, %)	9, 36.0	23, 28.4	0.467

*eGFR* Estimated glomerular filtration rate, *UPCR* Urine protein creatinine ratio, *ESR* Erythrocyte sedimentation rate, *CRP* C reactive protein, *MPO* Myeloperoxidase, *PR3* Proteinase 3, *BVAS* Birmingham Vasculitis Activity Score

### Predictors for response of intravenous methylprednisolone pulses

**Fig. 2:**
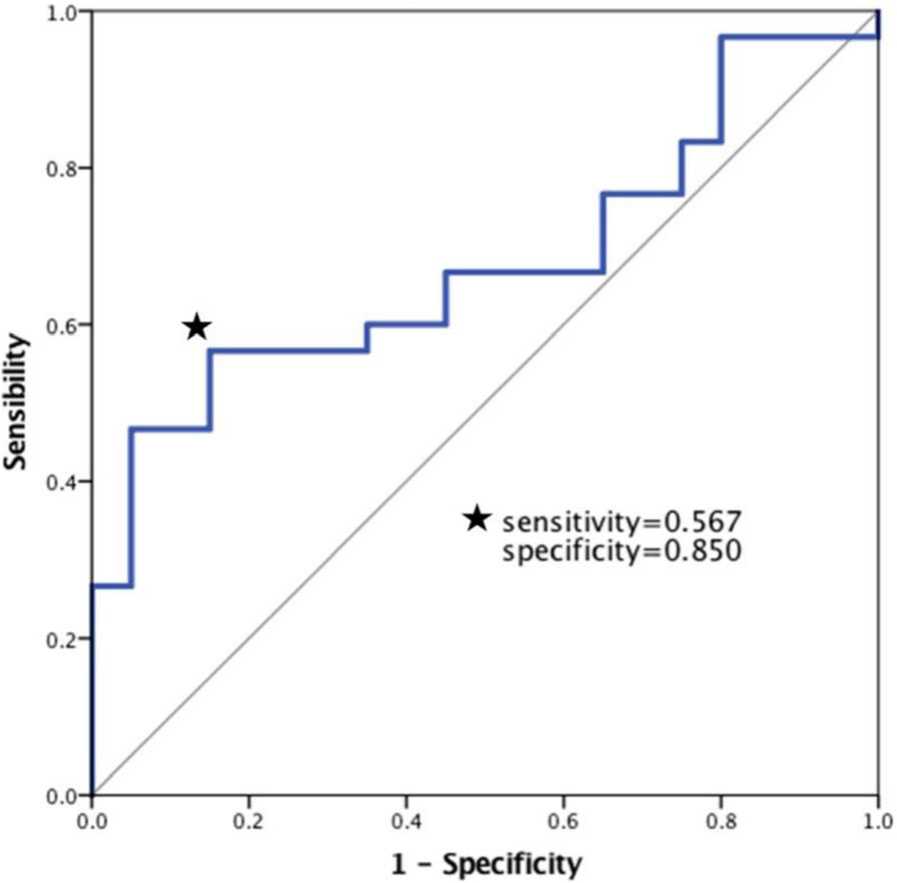
Receiver operating characteristic curve analysis using serum creatinine and urine protein creatinine ratio at baseline for prediction of death or maintaining dialysis in patients treated with intravenous methylprednisolone pulses.

We reclassified 53 patients in MP group (exclude 4 lost patients) into two groups according to the outcomes. Twenty-one patients with the outcome of dialysis independence were included in positive response group while 32 patients with the outcome of death or maintaining dialysis were included in negative response group. The data of these two groups were shown in Table 5. In the univariate analysis of clinical characteristics, we found the differences of serum creatinine level and UPCR level at admission reached the standard of P value less than 0.1. We used serum creatinine and UPCR in ROC analysis comparing negative response group with positive response group. The area under ROC curve was 0.678 (95% confidence interval, 0.530 to 0.826; *P* = 0.034), shown in Fig. 2. The cut-off values based on maximum value of Youden index were serum creatinine≥855μmol/L and UPCR≥3.7g/24h (sensibility 56.7%, specificity 85.0%, PPV 100.0% and NPV57.1%). We concluded that those patients with serum creatinine≥855μmol/L and UPCR≥3.7g/24h at admission may not have good responses to intravenous methylprednisolone pulses. A total of 63 adverse events were reported in positive response group and negative response group, shown in Table S2 in the supplemental file. The rates of adverse events were similar between two groups. Infections, thrombocytopenia and leukopenia were common in both groups.

**Table 5:** Univariate analysis of predictors for treatment responses of intravenous methylprednisolone pulses

Characteristics	Positive response group (*n* = 21)	Negative response group (*n* = 32)	*P* value
Age (year)	58.9 ± 12.7	56.2 ± 12.4	0.450
Female (n, %)	16, 76.2	21, 65.6	0.544
White blood cells (10^9^/L)	9.8 ± 4.8	8.3 ± 4.9	0.269
Hemoglobin (g/L)	73.7 ± 15.1	73.6 ± 11.7	0.984
Platelet (10^9^/L)	236.0 ± 107.3	195.8 ± 67.6	0.137
Serum albumin (g/L)	30.9 ± 4.9	31.1 ± 5.1	0.889
Serum globulin (g/L)	31.6 ± 7.1	29.9 ± 3.3	0.333
Alanine transaminase (u/L)	13.8 ± 13.3	14.3 ± 7.6	0.866
Serum creatinine (μmol/L)	607.6 ± 142.1	701.2 ± 232.4	0.073
eGFR (ml/min/1.73 m^2^)	7.1 ± 2.0	6.7 ± 1.9	0.461
UPCR (g/24 h)	2.5 ± 0.7	3.4 ± 2.2	0.040
ESR (mm/h)	88.4 ± 43.3	87.9 ± 35.9	0.963
CRP (mg/L)	55.5 ± 55.6	36.5 ± 45.3	0.179
MPO-ANCA postive (case, %)	18, 85.7	29, 90.6	0.671
PR3-ANCA postive (case, %)	4, 19.0	3, 9.4	0.415
Pulmonary involvement (case, %)	16, 76.2	24, 75.0	1.000
BVAS	14.9 ± 3.1	15.4 ± 3.0	0.559
Kidney length (cm)	10.7 ± 1.0	10.6 ± 1.1	0.798
Infections (case, %)	9, 42.9	12, 37.5	0.778

*eGFR* Estimated glomerular filtration rate, *UPCR* Urine protein creatinine ratio, *ESR* Erythrocyte sedimentation rate, *CRP* C reactive protein, *MPO* Myeloperoxidase, *PR3* Proteinase 3, *BVAS* Birmingham Vasculitis Activity Score

## Discussion

For induction therapy in AAV related renal injury, high-dose corticosteroids were suggested to use in combination with the other immunosuppressive medications (e.g. cyclophosphamide or rituximab) [24]. One study however linked the extended use of corticosteroids (>6 months) with an increased risk of infections without a significant decrease in the risk of disease relapse [25]. The rationale for pulse methylprednisolone was related to its rapid anti-inflammatory effects. High-dose methylprednisolone may also contribute to a rapid reduction in ANCA-producing plasma cells. The only randomized evaluation of pulse methylprednisolone (1000mg per pulse for 3 pulses) was in the setting of MEPEX trial, where it was compared to plasmapheresis as adjunctive therapy to oral corticosteroids and oral cyclophosphamide [18]. In that trial, pulse methylprednisolone was less efficacious than plasmapheresis in preserving kidney function. In this study, there were 6 patients receiving plasma exchange as we screened. Since six patients were not enough to be analyzed as a new group, we excluded them before entry. However, the value of pulse methylprednisolone as induction therapy had not been investigated directly. Besides, no published studies were specific to Chinese AAV patients with severe kidney involvement. Cohort studies did not detect a GFR level below which the therapy could be deemed futile, as remission occurred in about 57% of patients with a GFR of 10 ml/min/1.73 m^2^ or less at presentation [21]. Thus in patients with severe pauci-immune NCGN requiring dialysis, the question raised as whether the risks of intensive therapy were greater than the likelihood of recovering kidney function, and whether there was the factors that could predict the response to intensive treatment.

In the present study, we retrospectively included 111 Chinese AAV patients with severe renal involvement (eGFR<10ml/min/1.73m^2^). The results showed that the therapy of intravenous methylprednisolone 500mg/d for 3 days could improve the long-term outcome, in terms of dialysis independence in a median follow up period of 31 months in these patients. Furthermore, there was no difference in mortality (*P* = 0.056) and adverse events between groups. Patients with serum creatinine level less than 855μmol/L and UPCR less than 3.7g/24h at presentation may have better response to intravenous methylprednisolone pulses. It indicated that the cut-off levels of urine protein and serum creatinine could provide non-invasive estimate of the efficacy of intravenous methylprednisolone pulses. Our previous study showed that proteinuria higher than 3.5g/24h was one of the independent factors for worse renal outcome [26]. Bertha *et al* also found that proteinuria at presentation could predict renal survival, with a hazard ratio of 1.49 (confidence interval was 1.03 to 2.14, *P* = 0.034) [27]. So we suggested that UPCR and serum creatinine levels could be used to distinguish responders from non-responders for methylprednisolone pulses therapy and have important implications for treatment decisions in AAV patients with severe renal involvement.

This study had several limitations. First, all patients were Chinese; this population may have higher percentage of MPA, higher percentages of sclerotic and mixed lesions in renal pathology than western populations [7, 14]. Second, not all the patients had renal biopsy and the percentage of renal biopsies was not balance in two arms. Only 34 patients had kidney biopsy results (28 biopsies in MP group and 6 biopsies in control group) in this study, so renal histopathological analysis had questionable significance. One reason was that the ultrasonic kidney length was significant less in control group than that in MP group as we shown in Table 1. In the control group, patients with small sized kidney may have been considered as late referred patients with irreversible renal failure who should not have kidney histology and prednisolone pulses. However when we analyzed the characteristics in dialysis independent group and death or maintaining dialysis group, there were no differences on the rate of biopsy or kidney length between groups, and the Cox regression analysis found these parameters were not the independent risk factors. Third, for the retrospective nature, the maintaining immunosuppressive regimen in two groups were not unified, including full dose prednisone and half dose prednisone combined with intravenous cyclophosphamide or mycophenolate mofetil. These limitations may compromise the validity and utility of the present results. A well-designed, randomized, controlled study is needed to confirm the above results.

## Conclusions

Our study showed that for AAV patients with severe renal involvement, intravenous methylprednisolone pulses could improve the long-term outcome in term of dialysis independence and have a tendency of decreasing mortality. It encouraged use of appropriate intensive immunosuppressive therapy in certain population.

## Additional files

Additional file 1: The dataset analyzed in the present study. (XLSX 33 kb)

Additional file 2: Figure S1. The yearly distribution of patients in MP group and control group from 2004 to 2016. **Table S1.** The baseline pathological characteristics in MP group and control group. **Table S2.** Adverse events in MP group for treatment responses of intravenous methylprednisolone pulses. (DOCX 191 kb)

## Abbreviations

ANCA: Anti-neutrophil cytoplasmic autoantibody
AAV: Anti-neutrophil cytoplasmic autoantibody associated vasculitides
eGFR: Estimated glomerular filtration rate
GPA: Granulomatosis with polyangiitis
MPA: Microscopic polyangiitis
EGPA: Eosinophilic granulomatosis with polyangiitis
NCGN: Necrotizing and crescentic glomerulonephritis
ESRD: End-stage renal disease
KDIGO: Kidney Disease: Improving Global Outcomes
GBM: Anti-glomerular basement membrane
UPCR: Urine protein creatinine ratio
ESR: Erythrocyte sedimentation rate
CRP: C reactive protein
MPO: Myeloperoxidase
PR3: Proteinase 3
BVAS: Birmingham Vasculitis Activity Score
IQR: Interquartile range
