# Supplementary material for: The impact of intravenous methylprednisolone pulses on renal survival in anti-neutrophil cytoplasmic antibody associated vasculitis with severe renal injury patients: a retrospective study
Source: BMC Nephrol. 2017 Dec 29;18:381. doi: 10.1186/s12882-017-0782-4 (PMC5747949; doi:10.1186/s12882-017-0782-4)
Supplement: Supplementary file 2 — The yearly distribution of patients in MP group and control group from 2004 to 2016. Table S1. The baseline pathological characteristics in MP group and control group. Table S2. Adverse events in MP group for treatment responses of intravenous methylprednisolone pulses. (DOCX 191 kb) [file 12882_2017_782_MOESM2_ESM.docx]

Additional file 2


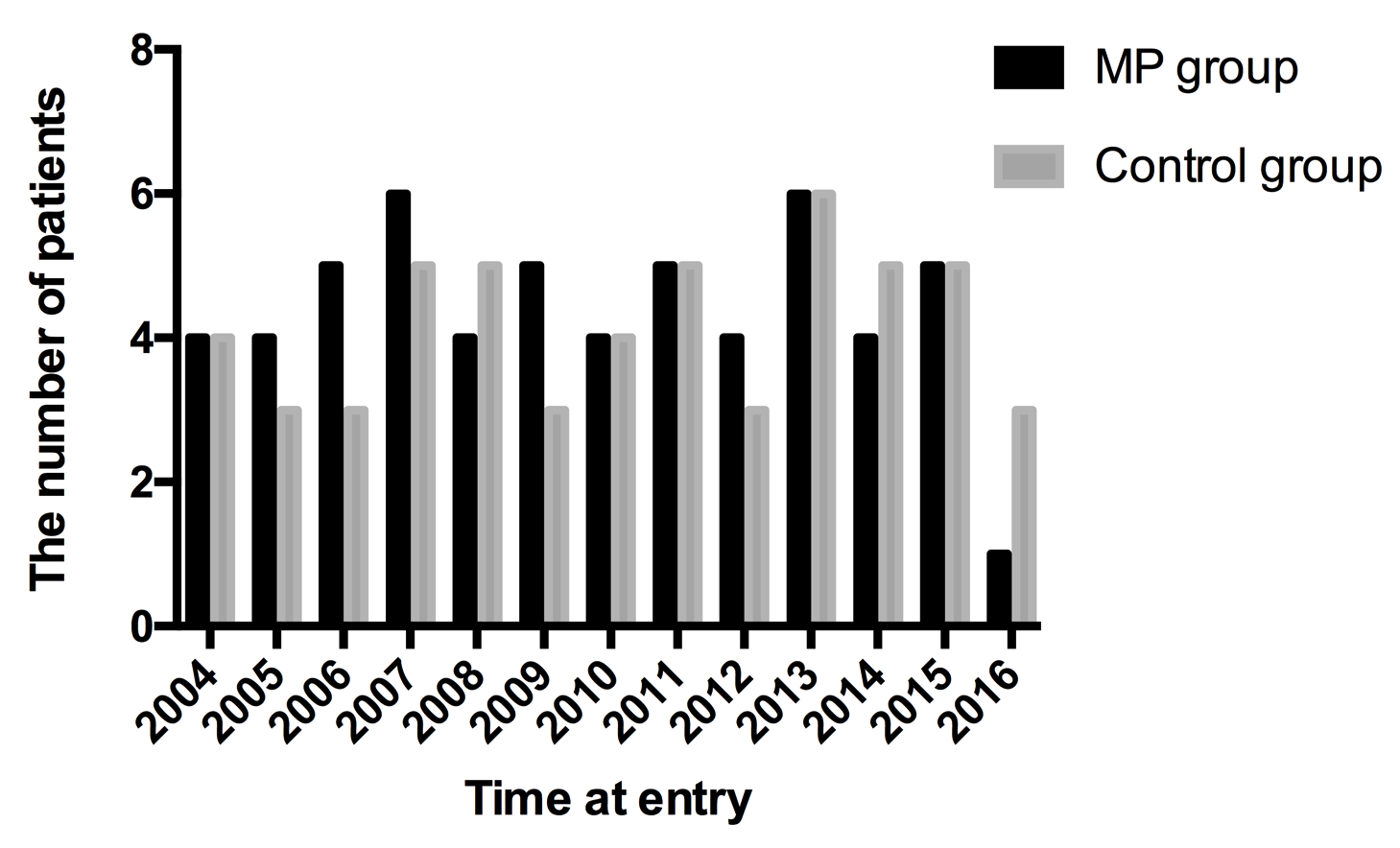


Figure S1. The yearly distribution of patients in MP group and control group from 2004 to 2016.

Table S1. The baseline pathological characteristics in MP group and control group.

|  | MP group  (n=28) | Control group  (n=6) | P value |
| --- | --- | --- | --- |
| Glomerular global sclerosis (median (IQR), %) | 33.0(14.3-64.4) | 47.9(29.2-84.4) | 0.653 |
| Cellular crescent (median (IQR), %) | 24.0(5.6-50.6) | 12.5(4.9-21.6) | 0.653 |
| Fibrinoid necrosis (case, %) | 8, 28.6 | 1, 16.7 | 0.644 |
| Mesangial proliferation 1/2/3 (case) | 3/8/17 | 0/3/3 | 0.843 |
| Interstitial infiltrates 0/1/2/3 (case) | 2/13/9/4 | 1/3/1/1 | 0.612 |

Table S2. Adverse events in MP group for treatment responses of intravenous methylprednisolone pulses.

| Adverse events | Positive response group  (n=21) | Negative response group  (n=32) | P value |
| --- | --- | --- | --- |
| Infection | 13 | 15 | 0.400 |
| bacteria | 9 | 10 | 0.559 |
| fungus | 3 | 1 | 0.289 |
| virus | 1 | 4 | 0.637 |
| Thrombocytopenia | 5 | 8 | 1.000 |
| Leukopenia | 4 | 4 | 0.698 |
| Diabetes | 3 | 1 | 0.289 |
| Gastrointestinal | 1 | 3 | 1.000 |
| Cardiovascular | 1 | 1 | 1.000 |
| Hemorrhage | 0 | 3 | 0.269 |
| Epilepsy | 1 | 0 | 0.396 |
| Totals | 28 | 35 |  |
